# Safety, Tolerability, and Pharmacokinetics of the Long‐Acting SARS‐CoV‐2–Neutralizing Monoclonal Antibody Combination AZD7442 (Tixagevimab/Cilgavimab) in Healthy Chinese Adults

**DOI:** 10.1002/cpdd.1583

**Published:** 2025-08-13

**Authors:** Nanyang Li, Jing Zhang, Wenhong Zhang, Zhongyuan Xu, Xiangcao Yao, Anqi He, Shuyuan Liu, Xiaoyun Ge, Jinxi Liu, Yunfei Li, Cecil Chi‐Keung Chen, Huixia Zhang

**Affiliations:** ^1^ Clinical Pharmacology Research Center Huashan Hospital Fudan University Shanghai China; ^2^ Clinical Trial Institution Clinical Pharmacology Research Center Huashan Hospital Fudan University Shanghai China; ^3^ Department of Infectious Diseases Huashan Hospital Fudan University Shanghai China; ^4^ Shanghai Sci‐Tech Inno Center for Infection & Immunity Shanghai China; ^5^ Clinical Pharmacy Center Nanfang Hospital Southern Medical University Guangzhou China; ^6^ Respiratory & Immunology R&D China AstraZeneca Shanghai China; ^7^ R&D China AstraZeneca Shanghai China; ^8^ Clinical Safety R&D China AstraZeneca Shanghai China; ^9^ Clinical Pharmacology and Safety Sciences BioPharmaceuticals R&D AstraZeneca South San Francisco CA USA; ^10^ Clinical Pharmacology and Quantitative Pharmacology Clinical Pharmacology & Safety Sciences BioPharmaceuticals R&D AstraZeneca Gaithersburg MD USA

**Keywords:** cilgavimab, clinical trial, COVID‐19, monoclonal antibody, tixagevimab

## Abstract

AZD7442, a combination of extended half‐life monoclonal antibodies tixagevimab and cilgavimab, was shown to neutralize previously circulating SARS‐CoV‐2 variants. This study evaluated safety, tolerability, pharmacokinetics, and pharmacodynamics of AZD7442 in healthy Chinese adults. In this randomized, placebo‐controlled, Phase 1 study, AZD7442 was administered intramuscularly or intravenously (300 or 600 mg). End points included safety, tolerability, pharmacokinetics, antidrug antibodies, and SARS‐CoV‐2–neutralizing antibody titers. Sixty participants were randomized and dosed (AZD7442, n = 49; placebo, n = 11). Adverse events occurred in 45 (91.8%) and 9 (81.8%) participants, serious adverse events occurred in 2 (4.1%) and 0 (0%) participants in AZD7442 and placebo groups, respectively, and there were no deaths. Tixagevimab and cilgavimab had mean half‐lives of 82.4‐88.1 (range across dosing groups) and 79.0‐83.7 days, respectively. In participants who received AZD7442, 3 (6.1%) were treatment‐emergent antidrug antibody positive. SARS‐CoV‐2–neutralizing antibody titers were more than 4‐fold higher than baseline levels by Day 8, then decreased through Day 361 following AZD7442 administration. AZD7442 was well tolerated in healthy Chinese adults, demonstrating predictable pharmacokinetics and an extended half‐life consistent with previous studies.

Although COVID‐19 is no longer considered a public health emergency, rates of severe disease, hospitalization, and death remain high, particularly among immunocompromised populations.^1^ The primary prophylactic approach against COVID‐19 is vaccination; however, the immunogenicity, type, and magnitude of immune responses generated by vaccines are often insufficient to protect against disease in immunocompromised individuals compared with the general population.^2^ Monoclonal antibodies (mAbs) represent an alternative option for immunocompromised individuals and those with contraindications to vaccination.^2^

AZD7442 is a combination of 2 long‐acting SARS‐CoV‐2–specific neutralizing mAbs: tixagevimab and cilgavimab.^3^ The progenitor mAbs were initially isolated from individuals with prior SARS‐CoV‐2 infection (Wuhan strain) and subsequently modified with 2 triple amino acid substitutions, YTE and TM, to extend serum half‐lives and reduce the theoretical risk of antibody‐dependent enhancement of disease, respectively.^3^ A global first‐in‐human (FIH) Phase 1 study (ClinicalTrials.gov: NCT04507256) investigating AZD7442 in healthy participants found the mAb pair to be well tolerated with a favorable safety profile across all intramuscular (IM; 300 mg) and intravenous (IV; 300‐3000 mg) doses.^4^ Additionally, predictable pharmacokinetic (PK) profiles were observed for both mAbs and SARS‐CoV‐2–specific neutralizing antibody (nAb) titers, which increased in a dose‐dependent manner in parallel with serum AZD7442 concentration and were maintained above the convalescent plasma level throughout the trial period.^4^ In Phase 3 studies, AZD7442 has demonstrated efficacy for the prevention and treatment of COVID‐19.^5^, ^6^ Recently, long‐term safety data from the PROVENT, STORM CHASER, and TACKLE Phase 3 studies reported that the safety of AZD7442 up to 15 months after dosing was consistent with the respective primary analyses, supporting the long‐term use of long‐acting mAbs to prevent COVID‐19.^7^, ^8^ Although AZD7442 lost activity against Omicron variants, including BQ1.1 and XBB,^9^, ^10^ it is still important to assess safety and PK in populations that were underrepresented in the global Phase 3 trials, as this may help to inform development of future long‐acting mAbs with YTE and TM modifications. This study evaluated the safety, tolerability, PK, anti‐drug antibody (ADA) responses, and pharmacodynamics (PD) of IM or IV AZD7442 administered to healthy Chinese participants.

## Methods

### Trial Design

This Phase 1, randomized, double‐blind, placebo‐controlled study evaluated the safety, tolerability, PK, and PD of single doses of AZD7442 in healthy Chinese adults aged 18‐55 years (ClinicalTrials.gov: NCT05437289). The study was conducted at 2 clinical study centers in mainland China (Table S1). The study was performed in accordance with the ethical principles that have their origin in the Declaration of Helsinki and that are consistent with the International Conference on Harmonization guidance, Good Clinical Practice, applicable regulatory requirements, and all applicable laws and regulations. The protocol was reviewed by the relevant institutional review board/ethics committee at the respective study sites (Table S1). All participants provided informed written consent.

The planned study duration was 479 days, and the screening period was up to 27 days (Days −28 through −2). Eligible study participants were institutionalized for 3 days, which included admission procedures (Day −1), randomization, and AZD7442/placebo administration (Day 1), and discharge procedures (Day 2). After AZD7442/placebo administration, participants were followed up for approximately 15 months (through Day 451) for safety monitoring and blood sample collection to evaluate PK, PD, and ADA responses. As the study was conducted during the COVID‐19 pandemic (before the emergence of Omicron and subvariants), measures were established to ensure minimal SARS‐CoV‐2 exposure for site staff as well as study participants. Participants were monitored for COVID‐19 symptoms and tested for SARS‐CoV‐2 if warranted, based on the investigator's judgment. When participants became eligible for the nationally deployed COVID‐19 vaccine and it was locally available, they could be unblinded upon request but remained in the study.

### Treatment Allocation and Blinding

Eligible participants were centrally assigned to the study intervention using a randomization and trial supply management system. Participants were randomized 4:1 to receive a single dose of either AZD7442 or placebo, administered intramuscularly or intravenously, across 4 fixed‐dose cohorts and respective placebo groups (300 mg IM or placebo, 600 mg IM or placebo, 300 mg IV or placebo, and 600 mg IV or placebo) (Figure S1). Placebo groups were pooled together for further analysis. Within each dose level, administration of AZD7442 and placebo was double‐blinded. However, due to the difference in dose volumes and administration routes, dose levels were not blinded. Investigators and participants remained blinded to the assigned dose until the end of the study.

### Study Treatments

Tixagevimab and cilgavimab were provided in separate vials as sterile, clear to opalescent, colorless to yellow solutions. The solutions contained 100 mg/mL of active ingredient (tixagevimab or cilgavimab) in 20 mM L histidine/L histidine hydrochloride, 240 mM sucrose, and 0.04% (w/v) polysorbate 80, at pH 6.0. Placebo was provided as a sterile solution of 0.9% (w/v) sodium chloride.

IM injections were administered via 2 sequential gluteal injections. The 300‐mg IM dose comprised 1 injection each of 1.5‐mL tixagevimab and 1.5‐mL cilgavimab. The 600‐mg IM dose comprised 1 injection each of 3‐mL tixagevimab and 3‐mL cilgavimab. Placebo was administered via 2 sequential gluteal injections of matching volumes.

For IV administration, tixagevimab and cilgavimab were added to a single IV bag and gently mixed before infusion. The 300‐mg IV infusion comprised 1.5 mL of tixagevimab plus 1.5 mL of cilgavimab; target infusion time was 15 minutes (20 mg/min). The 600‐mg IV infusion comprised 3 mL of tixagevimab plus 3 mL of cilgavimab; target infusion time was 30 minutes (20 mg/min). There was no specific guidance on the administration site for IV infusions, and this was not recorded, although it is typically the left or right hand in clinical practice.

### Inclusion and Exclusion Criteria

Participants were Chinese adults aged 18‐55 years with negative SARS‐CoV‐2 quantitative reverse transcription polymerase chain reaction and serology tests within 14 days before randomization. Participants had to be healthy by medical history, physical examination, baseline safety laboratory tests, and electrocardiogram (ECG) without clinically significant abnormalities at screening, according to the judgment of the investigator. Ineligible participants included those with known hypersensitivity or allergy to any AZD7442 dose component, or previous hypersensitivity, infusion‐related reaction, or severe adverse reaction to a mAb. Individuals were also excluded if they presented with acute illness (including fever greater than 37.8°C/100.0°F) on the day before or day of randomization. Any individual with immunodeficiency due to illness or medications, a history of SARS‐CoV‐2 infection or COVID‐19 symptoms 4 weeks before screening, or who had received a COVID‐19 vaccination was excluded. Full inclusion and exclusion criteria are included in the Supplemental Information.

### End Points

Safety and tolerability of IM or IV AZD7442 was assessed as adverse events (AEs), serious AEs (SAEs), AEs of special interest (injection site reactions [IM only], infusion‐related reactions [IV only], anaphylaxis, and other serious hypersensitivity reactions including immune complex disease), safety laboratory parameters, 12‐lead ECG, and vital signs. Also assessed were serum PK of AZD7442 (tixagevimab and cilgavimab) after a single dose of IM or IV AZD7442, ADA response to AZD7442 (tixagevimab and cilgavimab) in serum, and evaluation of neutralizing responses against SARS‐CoV‐2 in serum (PD parameter).

### Assessments

AEs and SAEs were reported by participants throughout the study period, either spontaneously or in response to questioning. After administration of AZD7442 on Day 1, participants were followed up for approximately 15 months for safety monitoring and blood sample collection for PK, treatment‐emergent (TE)‐ADA, and SARS‐CoV‐2–specific nAb evaluation on Days 4, 6, 8, 15, 31, 61, 91, 181, 271, and 361. ADA and nAb samples were not collected on all of the days that are listed. TE‐ADA status for tixagevimab or cilgavimab was defined as either treatment‐induced ADA positive or treatment‐boosted ADA positive. Treatment‐induced ADA positive was defined as ADA negative at baseline and at least 1 post‐baseline assessment that was ADA positive with an ADA titer at least 2‐fold of the respective minimum required dilution. Treatment‐boosted ADA positive was defined as ADA positive at baseline, where the baseline titer was boosted by at least 4‐fold at 1 or more post‐baseline time points during the study period.

### Analysis of Tixagevimab and Cilgavimab Serum Concentrations

Serum tixagevimab and cilgavimab concentrations in clinical samples were measured by Labcorp Pharmaceutical Research and Development Co. Ltd. (Shanghai, China), with an immunoaffinity approach using streptavidin magnetic beads coated with biotinylated SARS‐CoV‐2 receptor binding domain (RBD) followed by liquid chromatography‐tandem mass spectrometry (LC‐MS/MS) in human serum. The method was validated per regulatory guidance and methods were previously reported.^11^ Human serum samples were prepared by diluting 20 µL of sample with 180 µL of Tris(hydroxymethyl)‐aminomethane‐buffered saline and TBS‐Tween‐20/bovine serum albumin buffer and mixing with 25 µL of high‐capacity Magne streptavidin beads (Promega Corporation) precoated with biotinylated RBD. The captured proteins were subjected to standard protein denaturation, reduction, alkylation, and on‐bead digestion with trypsin to produce characteristic peptides that were quantified as surrogates for tixagevimab and cilgavimab in the human serum samples. The digested samples were mixed with internal standard, then quenched with acid and passed through a multiscreen filter plate before injection on to the LC‐MS/MS for detection. Tixagevimab and cilgavimab reference standards and SARS‐CoV‐2 RBD were provided by AstraZeneca. Stable isotope labeled internal standard peptides, ASGF‐IS (ASGFTFMSSAVQWVR*, R* = ^13^C_6_, ^15^N^4^) for tixagevimab, and DVWM‐IS (DVWMSWVR*) for cilgavimab, were supplied by Elim Biopharmaceuticals. LC‐MS/MS used an LC PAL autosampler (CTC Analytics AG) with Agilent pumps and column heater (mobile phase A: 100:0.1 water/formic acid, mobile phase B: 100:0.1 acetonitrile/formic acid, flow rate: 0.35 to 0.45 mL/min) with SCIEX 6500+ (low‐mass mode, positive polarity): tixagevimab, transition monitored: m/z 837.4 to m/z 932.5, DP: 80, EP: 10, CE: 42; cilgavimab, transition monitored: m/z 539.7 to m/z 864.5, DP: 40, EP: 10, CE: 30. Data were analyzed with SCIEX Analyst software. The validated range of the assay is 0.3 µg/mL (lower limit of quantification) to 30.0 µg/mL (upper limit of quantification).

### Analysis of SARS‐CoV‐2 nAbs

SARS‐CoV‐2 nAbs in human clinical serum samples were measured using the PhenoSense SARS‐CoV‐2 neutralizing antibody assay (Monogram Biosciences), which is based on previously described assays using HIV‐1 pseudovirions,^12^, ^13^ and has been validated for analysis of SARS‐CoV‐2 nAbs.^14^ Assays were performed by Monogram.

Briefly, HEK293 cells were transfected with an HIV genomic vector containing a luciferase reporter gene plus an envelope expression vector carrying the SARS‐CoV‐2 spike protein open reading frame. Pseudovirions were collected and incubated with serial dilutions of clinical serum samples and HEK293 cells expressing the angiotensin‐converting enzyme 2 receptor. nAb activity was measured as the inhibition of luciferase activity in these cells as previously reported.^15^, ^16^

### Analysis of ADAs

Clinical samples were screened for ADAs against tixagevimab and cilgavimab using validated Meso Scale Discovery (MSD)‐based electrochemiluminescence (ECL) assays by Labcorp Pharmaceutical Research and Development Co. Ltd. The method involves 3 tiers of testing: screening assay (to screen out potentially positive samples), confirmation assay (to assess specificity of the potentially positive samples identified in the screening assay), and titration assay (to estimate level of antibody from confirmed positive samples). Samples were stored frozen at −60 to −80°C and thawed to room temperature before use. Positive controls were pooled human serum spiked with anti‐tixagevimab or anti‐cilgavimab antibodies (provided by AstraZeneca); negative controls were pooled human serum. Positive controls were prepared at low, medium, and high concentrations (6, 100, and 2500 ng/mL for anti‐tixagevimab and 10, 100, and 2000 ng/mL for anti‐cilgavimab). Human serum samples (and positive and negative controls) were prepared by diluting 1/20 with assay buffer (LowCross‐Buffer, CANDOR Bioscience) and incubated for 5‐10 minutes at room temperature with shaking at 600 rpm. Diluted samples (75 µL) were transferred to new dilution plate wells, with 75 µL of screen/titer solution (2 µg/mL of biotinylated tixagevimab or cilgavimab [provided by AstraZeneca] plus 2 µg/mL of ruthenium [sulfo‐tag]‐labeled tixagevimab or cilgavimab [provided by AstraZeneca] in assay buffer) or confirm solution (20 µg/mL of tixagevimab or cilgavimab, 2 µg/mL of biotinylated tixagevimab or cilgavimab plus 2 µg/mL of ruthenium [sulfo‐tag]‐labeled tixagevimab or cilgavimab in assay buffer) and incubation for 60‐90 minutes with shaking at 600 rpm. Streptavidin‐coated MSD plates were blocked by adding 150 µL of Blocker Casein in PBS (Thermo Fisher Scientific) per well and incubated for 1‐3 hours with shaking at 600 rpm, then washed 4 times with wash buffer (1X phosphate‐buffered saline, 0.05% Tween‐20). Next, 50 µL of the screen, titer, or confirmation samples (or positive or negative controls) were added to the MSD plates, which were then incubated for 60‐90 minutes at room temperature at 600 rpm. Finally, the plate was washed 4 times with 350 µL wash buffer, and then 150 µL Read Buffer T (2X) (MSD) was added to each well of the MSD plate. MSD plates were read within 15 minutes on a MESO Quickplex 120 plate reader (MSD). Samples were reported screen positive for ADA in the screening assay if the mean ECL value was at or above the ECL value of the plate‐specific cut point. Plate‐specific cut points were derived as median negative control response multiplied by the normalization factor (1.38 for the tixagevimab assay and 1.21 for the cilgavimab assay). Screen‐positive samples were retested in a confirmation assay, where samples were analyzed both in the absence and presence of excess drug to determine if the sample's positive response was specific to tixagevimab or cilgavimab. Samples generating a percentage inhibition value equal to or greater than the confirmatory cut point established for the method were reported as confirmed positive for ADA. Titers were measured in confirmed positive samples and were reported as the reciprocal of the highest 2‐fold dilution that measured positive in the assay, before returning a negative response. Titers for negative samples were reported as less than 80 for tixagevimab or less than 40 for cilgavimab since the minimum required dilution of the assay is 1:80 and 1:40, respectively.

### Statistical Analyses

No sample size calculation was performed on the basis of hypothesis testing, and descriptive statistics were used for the key end point assessments. The safety analysis set included all participants who received any dose of AZD7442 or placebo. The PK analysis set included all participants in the safety analysis set who received AZD7442 and had evaluable serum PK data, with no important protocol deviations considered to impact the analysis of the PK data. AZD7442 serum concentrations represent the arithmetic sum of tixagevimab and cilgavimab concentrations. Serum concentration values below the lower limit of quantification were set to 0 for inclusion in the calculation of means. Serum PK in healthy Chinese participants was compared graphically with data observed in the global FIH Phase 1 study of AZD7442,^4^ and PK parameters were compared between the 2 studies.

## Results

### Participants

The first participant was enrolled on October 9, 2021. The first participants were dosed on October 14, 2021, and the last participants were dosed on October 29, 2021. The final participant completed the study on January 16, 2023. Overall, 256 participants were screened, and 61 participants were randomized: 12 were randomized to each cohort except for the 600‐mg IV cohort, to which 13 participants were randomized (Figure S1). Of the 61 participants randomized, 60 participants received either AZD7442 or placebo and completed the study. One participant withdrew from the placebo cohort due to an AE (Figure S1). All treated participants were included in the safety analysis set. Forty‐nine participants who received AZD7442 were included in the PK analysis set. Demographics and key participant characteristics are summarized in Table S2. Similar baseline demographics were observed across the AZD7442 and placebo groups. The mean participant age was 32.0 and 31.0 years for the AZD7442 and placebo groups, respectively. The mean body weight was 64.4 kg for the pooled AZD7442 cohorts and 67.2 kg for the pooled placebo group.

### Safety of AZD7442

By Day 451, 45 (91.8%) participants receiving AZD7442 had AEs (Table S3). In the pooled placebo group, 9 (81.8%) participants reported AEs. Most AEs were mild in severity, with 75.5% in the pooled AZD7442 group and 72.2% in the pooled placebo group experiencing mild AEs. The most commonly reported AEs in the total AZD7442 group and pooled placebo group, respectively, were COVID‐19 (24 [49.0%] and 3 [27.3%] participants), upper respiratory tract infection (12 [24.5%] and 3 [27.3%]), and fatigue (7 [14.3%] and 1 [9.1%]). There were 30 participants with COVID‐19–related AEs (27 [55.1%] participants who received AZD7442, and 3 [27.3%] participants who received placebo), including those confirmed with a positive antigen or nucleic acid test result, suspected COVID‐19, and asymptomatic COVID‐19. Of these individuals, 29 experienced the events late in the study, occurring from Day 364 onward. One participant in the AZD7442 600‐mg IM group had an asymptomatic COVID‐19 AE that started on Day 173. Two participants had SAEs that were not considered related to AZD7442. One participant in the AZD7442 600‐mg IM group reported an SAE of appendicitis and 1 participant in the AZD7442 600‐mg IV group had an SAE of inguinal hernia. One participant had an AE of special interest (injection‐site erythema) in the AZD7442 600‐mg IM group. No infusion‐related reactions, anaphylaxis, or other serious hypersensitivity reactions were observed. There were no deaths or AEs leading to dosing discontinuation, interruption, or withdrawal from the study. There were no clinically meaningful trends between the treatment groups in clinical chemistry, clinical hematology, coagulation, urinalysis, mean changes in vital sign or ECG parameters, or in the shifts from baseline.

### PK of Tixagevimab and Cilgavimab in Serum

**Figure 1:**
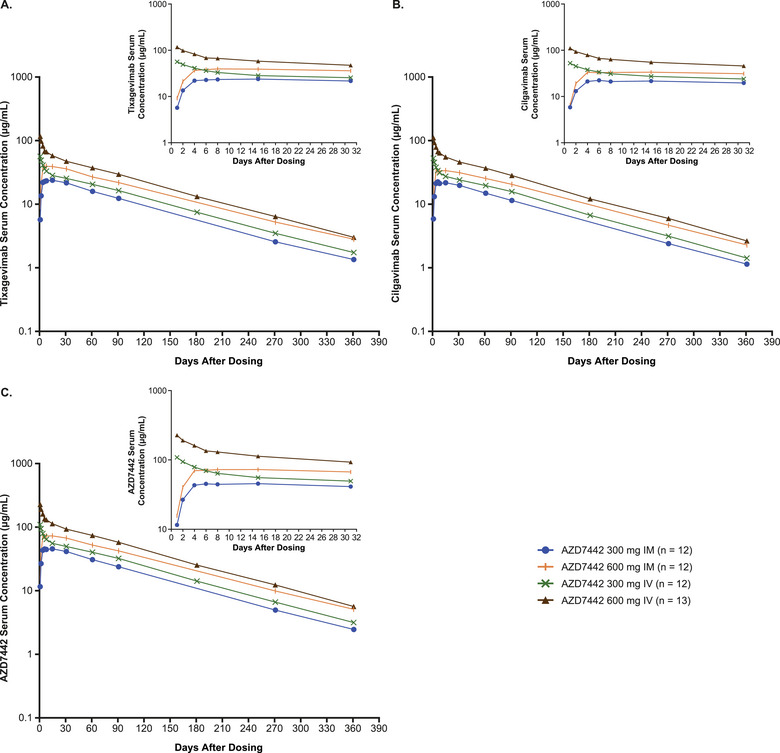
Serum concentration of (A) tixagevimab, (B) cilgavimab, and (C) AZD7442 (tixagevimab/cilgavimab) following a single IM or IV dose administration of AZD7442 in healthy Chinese adults. Data are presented as arithmetic means. Nominal sampling elapsed time (ie, time difference between nominal sampling time and dosing time) is used. Serum concentration values below the lower limit of quantification (0.3 µg/mL) were set to 0 for inclusion in the calculation of means. AZD7442 serum concentrations represent the arithmetic sum of tixagevimab and cilgavimab concentrations. If 1 or more component is BQL, then AZD7442 is BQL. BQL, below the quantification limit; IM, intramuscular; IV, intravenous.

**Figure 2:**
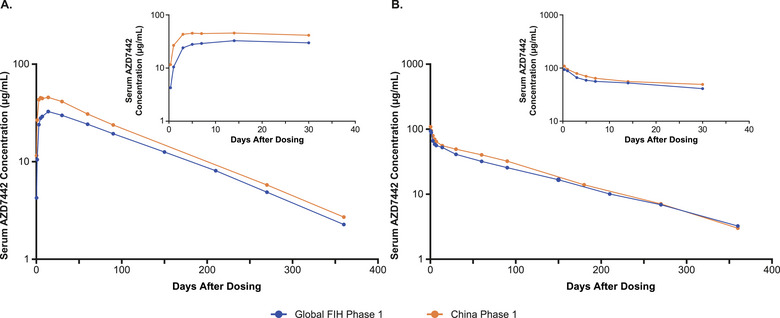
Comparison of AZD7442 serum concentration‐time profiles following a single 300‐mg (A) intramuscular injection and (B) intravenous infusion in healthy volunteers between global Phase 1 study and China Phase 1 study. Data are presented as arithmetic means. Global FIH data are adapted from Forte‐Soto. *J Infect Dis*. 2023.^4^ FIH, first‐in‐human.

Tixagevimab and cilgavimab exhibited extended arithmetic mean half‐lives of 82.4‐88.1 days and 79.0‐83.7 days, for 300‐ and 600‐mg IM doses, respectively, in healthy Chinese participants similar to the half‐lives reported in the global FIH Phase 1 study (Table 1). There was reduced availability of the mAbs following IM administration relative to IV (indicated by bioavailability), because some of the IM dose may bind to tissue components that reduce the fraction absorbed, and it may also be degraded by proteolytic enzymes before it reaches the systemic circulation. Arithmetic mean serum concentration‐time profiles for tixagevimab, cilgavimab, and AZD7442 following single doses of AZD7442 are shown in Figure 1A–C. Following IV infusion, the mean serum concentrations of tixagevimab and cilgavimab rapidly achieved the maximum values (58.4 and 118 µg/mL for tixagevimab 300 and 600 mg, respectively, and 55.1 and 112 µg/mL for cilgavimab 300 and 600 mg, respectively). Thereafter, serum concentrations slowly declined and remained quantifiable until 360 days after the single dosing. The serum concentration‐time profile of AZD7442 in healthy Chinese participants was consistent with observations from the global FIH study (Figure 2), though a higher exposure was noted for the China Phase 1 study. Following IM administration, the median time to maximum concentration for tixagevimab and cilgavimab was 7 and 6 days, respectively, for the 300‐mg dose cohort, and 14 and 7 days, respectively, for the 600‐mg dose cohort. Following IM administration, mean maximum concentration (C_max_), area under the concentration‐time curve (AUC) from time 0 to the time of last quantifiable concentration (AUC_last_), and AUC from time 0 extrapolated to infinity (AUC_inf_) for tixagevimab and cilgavimab increased as the AZD7442 dose increased from 300 to 600 mg; a 2‐fold increase in dose resulted in increases in C_max,_ AUC_last_, and AUC_inf_ of approximately 1.6‐1.8‐fold. The elimination phases of the serum concentration‐time profiles were parallel and similar across AZD7442 dose levels and between IM and IV administration. Within each administration route, serum concentration and total systemic exposure increased in an approximately dose‐proportional manner across the dose range for AZD7442.

**Table 1:** Serum PK Parameters of Tixagevimab and Cilgavimab Following a Single IM or IV Dose Administration of AZD7442 in Healthy Volunteers from China Phase 1 Study and Global Phase 1 Study (PK Analysis Set)

		Healthy Chinese participants (present study)				Global population (FIH Phase 1 study)^4^	
PK parameter	mAb	300 mg IM n = 12	600 mg IM n = 12	300 mg IV n = 12	600 mg IV n = 13	300 mg IM n = 10	300 mg IV n = 10
C_max_ (µg/mL)	Tixagevimab	24.9 (5.6)	41.6 (12.4)	58.4 (7.6)	118.3 (9.5)	17.4 (5.5)	54.0 (5.5)
	Cilgavimab	23.5 (5.4)	37.9 (11.0)	55.1 (8.4)	111.6 (11.0)	16.1 (4.9)	52.0 (6.3)
t_max_ (days)	Tixagevimab	7.0 (3.0, 29.0)	14.0 (3.0, 29.0)	0.2 (0.0, 0.3)	0.3 (0.0, 0.3)	14.0 (3.1, 30.0)	<0.1 (0.0, 0.3)
	Cilgavimab	6.0 (3.0, 29.0)	7.0 (3.0, 29.0)	0.2 (0.0, 1.0)	0.3 (0.0, 0.3)	14.0 (3.1, 60.2)	<0.1 (0.0, 1.0)
AUC_last_ (µg•day/mL)	Tixagevimab	2917 (574.9)	5208 (1483)	3849 (698.0)	7109 (880.0)	2451 (653.8)	3494 (478.6)
	Cilgavimab	2674 (543.6)	4676 (1284)	3594 (620.9)	6760 (856.7)	2094 (540.9)	3109 (424.3)
AUC_inf_ (µg•day/mL)	Tixagevimab	3087 (635.0)	5588 (1692)	4074 (830.3)	7478 (1007)	2621 (717.7)	3709 (526.7)
	Cilgavimab	2819 (592.3)	4965 (1428)	3767 (704.0)	7072 (961.4)	2211 (569.1)	3306 (491.5)
CL/(F) (L/day)	Tixagevimab	0.05091 (0.01259)	0.05821 (0.01664)	N/A	N/A	0.06174 (0.01857)	N/A
	Cilgavimab	0.05574 (0.01338)	0.06483 (0.01739)	N/A	N/A	0.07383 (0.02733)	N/A
V_z_ (L)	Tixagevimab	N/A	N/A	4.5 (0.6)	4.8 (0.4)	N/A	5.2 (0.6)
	Cilgavimab	N/A	N/A	4.6 (0.6)	4.9 (0.5)	N/A	6.0 (0.6)
t_1/2λz_ (days)	Tixagevimab	82.4 (14.1)	88.1 (13.1)	83.3 (13.9)	82.4 (9.7)	88.6 (12.7)	87.1 (4.5)
	Cilgavimab	79.0 (13.7)	83.7 (11.1)	79.6 (11.6)	79.1 (8.9)	80.1 (7.6)	91.4 (8.2)
F (%)	Tixagevimab	75.8	74.7	N/A	N/A	70.7	N/A
	Cilgavimab	74.8	70.2	N/A	N/A	66.9	N/A

Values are presented as arithmetic mean (standard deviation), except for t_max_, presented as median (minimum, maximum), and F, calculated as the single ratio of AUC_inf_ after IM to IV; thus, no measure of variation is shown.

AUC_inf_, area under the concentration‐time curve from time 0 extrapolated to infinity; AUC_last_, area under the concentration‐time curve from time 0 to the time of last quantifiable concentration; CL, clearance (IV administration only); CL/(F), apparent clearance after extravascular administration (IM administration only); C_max_, maximum concentration; F, bioavailability; FIH, first‐in‐human; IM, intramuscular; IV, intravenous; mAb, monoclonal antibody; N/A, not applicable; PK, pharmacokinetic; t_1/2λz_, terminal half‐life; t_max_, time to maximum concentration (IM administration only); V_z_, volume of distribution based on terminal phase after IV administration.

### SARS‐CoV‐2–nAb Titers and Serology

**Figure 3:**
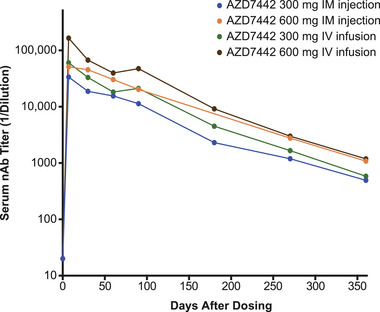
Serum nAb titers against SARS‐CoV‐2 following AZD7442 administration in healthy Chinese participants. Data are presented as geometric means. nAb concentrations per cohort for baseline (Day 0), Day 8, Day 181, and Day 361 visits. The Day 181 visit for the IM cohort was impacted due to Shanghai lockdown from March to June 2022. Therefore, Day 181 results were not available for most participants in the IM cohorts. IM, intramuscular; IV, intravenous; nAb, neutralizing antibody.

No participants had detectable nAbs before administration of AZD7442, and all participants receiving AZD7442 exhibited greater than 4‐fold increases in nAb titer compared with baseline at Day 8, with titers then decreasing through Day 361 (Figure 3).

One (1.7%) participant in the AZD7442 600‐mg IM group reported a positive response for SARS‐CoV‐2 antibodies at the Day 181 and 361 evaluations.

### ADAs to AZD7442

Among the participants who received AZD7442, ADA incidence (percentage TE‐ADA positive) was 4.1% (2 participants) for tixagevimab, 4.1% (2 participants) for cilgavimab, and 6.1% (3 participants) for AZD7442. All 3 TE‐ADA‐positive participants were classified as persistently ADA positive (Table S4). The individual AUC_last_ and C_max_ values of the TE‐ADA‐positive participants were similar to the geometric mean of participants in the respective treatment groups, indicating that there were no apparent impacts on PK (data not shown). In the pooled placebo group, there were 2 ADA‐positive participants; 1 (9.1%) had positive results to tixagevimab only, and 1 (9.1%) had positive results to cilgavimab only. There were no TE‐ADA‐positive participants in the placebo group.

## Discussion

This study demonstrated that a single dose of AZD7442 (300 or 600 mg, via IM or IV administration) was well tolerated in healthy Chinese adults over 450 days following dosing, with no notable differences in the overall safety profile, as observed in previous study populations.^4^, ^5^, ^6^, ^17^ There were no deaths and no AEs leading to discontinuation or interruption of AZD7442 or withdrawal from the study. COVID‐19‐related AEs occurred in 27 (55.1%) participants who received AZD7442, and 3 (27.3%) participants who received placebo; however, due to small numbers, no inference can be drawn from comparing the groups. Prior data suggested that AZD7442 could provide protection against COVID‐19 for up to approximately 6 months after dosing.^4^, ^5^ Most COVID‐19‐related AEs occurred late in the study, with 29 of 30 (96.7%) occurring from Day 364. These cases occurred after COVID‐19 restrictions were lifted in China in December 2022, by which time AZD7442 would not be expected to provide protection. The PK profile of AZD7442 and the individual mAbs in this study were as expected and consistent with those seen in global and Japanese studies.^3^, ^4^, ^17^ PK exposure increased almost dose proportionally from 300 to 600 mg AZD7442 with both IM and IV administration. AZD7442 serum PK profiles and the half‐lives of tixagevimab and cilgavimab in healthy Chinese participants were similar to those seen in the global population from the FIH Phase 1 study,^4^ although a higher exposure was observed in the China Phase 1 study. This higher exposure after the same dose and route of administration could be due to the lower body weight (mean, 64.4 kg) of the study participants in the China study; while in the global FIH study,^4^ the mean body weight was 75.5 and 72.2 kg for the 300‐mg IM and 300‐mg IV cohorts, respectively. The finding is consistent with the population PK analysis of AZD7442 where body weight was found to have a statistically significant effect on AZD7442 clearance and volume, with heavier participants having a lower exposure due to higher clearance and a larger volume of distribution.^18^ However, the difference in the observed exposure was not considered clinically significant, and the same dose of AZD7442 was approved for use in individuals weighing at least 40 kg.

nAb titers increased in all participants who received AZD7442, which is consistent with global studies.^3^, ^4^ Furthermore, nAb titer levels remained above those of 28 individual convalescent plasma samples, measured in the same assay across all doses and time points assessed.^3^ Although several participants reported COVID‐19‐related AEs, these occurred late in the study as noted above, mostly after the last assessment for SARS‐CoV‐2 antibodies occurred on Day 361. Three participants who received AZD7442 had TE‐ADAs and were persistently ADA positive. ADA titers to AZD7442 in TE‐ADA–positive participants were generally low and did not appear to impact PK or safety outcomes for these participants. TE‐ADA occurred in a similar proportion of participants who received AZD7442 in the TACKLE Phase 3 study (5%)^6^ and the global FIH study (2%).^4^ A low number of patients in the placebo group were ADA positive. These cases were likely false positives due to the sensitivity of the assay.^4^ Real‐world data from China have indicated that AZD7442 is efficacious at preventing COVID‐19 caused by the dominant Omicron variants,^19^ including BA1, BA2, BA4, and BA5, in immunocompromised individuals,^20^ supporting the approach of using long‐acting mAbs for preventing COVID‐19.

Strengths of this study include its double‐blind, randomized design, and the investigation of different doses and administration routes using a comprehensive set of PK assessments. Limitations include the relatively small size of the study population and that most participants were male.

## Conclusions

This Phase 1 study in healthy Chinese adults showed that AZD7442 was well tolerated, with similar extended half‐life and PK profile of AZD7442 to the global FIH Phase I study.^4^ Furthermore, this study adds to the growing body of evidence showing that AZD7442 safety and PK are consistent in different populations, which may help inform the development of future long‐acting mAbs against COVID‐19.

## Author Contributions

All authors meet the ICMJE authorship criteria.

## Conflicts of Interest

The authors declare the following financial interests/personal relationships which may be considered as potential competing interests: N.L., J.Z., Z.X., X.Y., and W.Z. received investigator fees from AstraZeneca for the conduct of the study. All other authors are employees of, and may hold stock and/or stock options in, AstraZeneca.

## Funding

This work was supported by AstraZeneca.

## Supporting information



Supporting Information

## Data Availability

Data underlying the findings described in this manuscript may be requested in accordance with AstraZeneca's data‐sharing policy described at https://astrazenecagrouptrials.pharmacm.com/ST/Submission/Disclosure. AstraZeneca Group of Companies allows researchers to submit a request to access anonymized participant‐level clinical data, aggregate clinical or genomics data (when available), and anonymized clinical study reports through the Vivli web‐based data request platform.
