# Supplementary material for: Safety, Tolerability, and Pharmacokinetics of the Long‐Acting SARS‐CoV‐2–Neutralizing Monoclonal Antibody Combination AZD7442 (Tixagevimab/Cilgavimab) in Healthy Chinese Adults
Source: Clin Pharmacol Drug Dev. 2025 Aug 13;14(11):836–45. doi: 10.1002/cpdd.1583 (PMC12583985; doi:10.1002/cpdd.1583)
Supplement: Supplementary file 1 — Supporting Information [file CPDD-14-836-s001.pdf]

Supplemental Information

Eligibility Criteria

Inclusion Criteria

Age

Chinese adults aged 18–55 years inclusive, at the time of signing the informed consent.

Informed Consent

Capable of giving signed informed consent, which includes compliance with the requirements and restrictions listed in the informed consent form and study protocol.

Type of Participant

Healthy by medical history, physical examination, and baseline safety laboratory tests, according to the judgment of the investigator.

Negative results of both SARS-CoV-2 quantitative reverse transcription-polymerase chain reaction and serology tests within 14 days before randomization.

Able to complete the follow-up period through Day 451.

Electrocardiogram without clinically significant abnormalities at screening.

Weight

Body weight ≥50 kg to ≤110 kg and body mass index ≥18.0 to ≤30.0 kg/m^2 at the screening visit.

Reproduction

Contraceptive use by males or females was consistent with local regulations regarding the methods of contraception for those participating in clinical studies.

Contraceptive use by males or females:

- Male participants: To avoid transfer of fluids to a sexual partner, all male participants were to use a condom from Day 1 and agree to continue through 365 days following administration of the study intervention.
- Female participants: Female participants of childbearing potential were to use one highly effective form of birth control from Day 1 and agree to continue through 365 days following administration of the study intervention. Cessation of contraception after this point was to be discussed with a responsible physician. Periodic abstinence (calendar, symptothermal, post-ovulation methods), withdrawal (*coitus interruptus*), spermicides only, and lactational amenorrhea method were not acceptable methods of contraception. Female condom and male condom were not to be used together. All individuals of childbearing potential were to have a negative serum pregnancy test result at Visit 1 and throughout the study as indicated per the study schedule of activities.

Individuals not of childbearing potential were defined as those who were either permanently sterilized (hysterectomy, bilateral oophorectomy, or bilateral salpingectomy), or who were postmenopausal. Individuals were considered postmenopausal if they had been amenorrhoeic for 12 months prior to the planned date of randomization without an alternative medical cause. The following age-specific requirements applied:

- Individuals <50 years of age were considered postmenopausal if they had been amenorrhoeic for 12 months or more following cessation of exogenous hormonal treatment and follicle stimulating hormone levels in the postmenopausal range.

- Individuals ≥50 years of age were considered postmenopausal if they had been amenorrhoeic for 12 months or more following cessation of all exogenous hormonal treatment.

A highly effective method of contraception was defined as one that could achieve a failure rate of <1% per year when used consistently and correctly.

Exclusion Criteria

Any of the following were regarded as a criterion for exclusion from the study:

Medical Conditions

Known history of allergy or reaction to any component of the study intervention formulation.

Previous hypersensitivity, infusion-related reaction, or severe adverse reaction following administration of a monoclonal antibody (mAb).

Significant infection or other acute illness, including fever >100°F (>37.8°C) on the day before, or day of, randomization. Participants excluded for transient acute illness could be dosed if the illness resolved within the 27-day screening period. Otherwise, the participant was to be reported as a screen failure. Upon obtaining an informed consent again, the participant could be rescreened just once.

History of infection with severe acute respiratory syndrome or Middle East respiratory syndrome.

History of laboratory-confirmed SARS-CoV-2 infection or any positive SARS-CoV-2 result based on available data at screening.

Any clinical signs and symptoms consistent with COVID-19 (eg, fever, dry cough, dyspnea, sore throat, fatigue, or confirmed infection by appropriate laboratory test within the last 4 weeks before screening or on admission).

History of Malignancy

History of clinically significant bleeding disorder (eg, factor deficiency, coagulopathy, or platelet disorder), or history of significant bleeding or bruising following intramuscular injections or venipuncture.

Either history of active infection with hepatitis B or C or positive test for hepatitis C or for hepatitis B surface antigen at screening.

Immunodeficiency due to illness, including human immunodeficiency virus (HIV) infection, or due to drugs, including any course of glucocorticoid therapy exceeding 2 weeks of prednisone or equivalent at a dose of 20 mg daily or every other day within 6 months before screening. HIV testing was to be negative at screening.

History of alcohol or drug abuse within the previous 2 years that, according to the investigator, might have affected assessments of safety or ability of the participant to comply with all study requirements, or a positive urine drug or alcohol screening.

Any other significant disease, disorder, or finding that may significantly increase the risk to the participant due to participation in the study, affect the ability of the participant to participate in the study, or impair interpretation of the study data.

Laboratory Abnormalities

Any of the following laboratory abnormalities at screening:

- Aspartate aminotransferase or alanine aminotransferase > upper limit of normal (ULN), or alkaline phosphatase or total bilirubin >1.5 × ULN.
- Serum creatinine > ULN.
- Hemoglobin < lower limit of normal (LLN).
- Platelet count < LLN.
- White blood cell or neutrophil count outside normal reference ranges.

Any other laboratory value in the screening panel that, in the opinion of the investigator, was clinically significant or might have confounded analysis of study results.

Prior/Concomitant Therapy

Any drug therapy within 7 days or 5 drug half-lives (whichever is longer) before Day 1 (except contraceptives or a single use of acetaminophen, aspirin, antihistamine, or combination over-the-counter [OTC] product that contains acetaminophen with an antihistamine, or OTC nonsteroidal anti-inflammatory agent at a dose equal to or lower than that recommended on the package). Vitamins and other nutritional supplements that were not newly introduced, ie, had been taken for ≥30 days before screening, were not exclusionary.

Receipt of immunoglobulin or blood products within 6 months before screening.

Any prior receipt of investigational or licensed vaccine or other mAb/biologic indicated for the prevention of SARS-CoV-2 or COVID-19 or scheduled receipt.

Receipt of a mAb within 6 months or 5 antibody half-lives (whichever is longer) before screening.

Prior/Concurrent Clinical Study Experience

Receipt of any investigation product within 90 days or 5 antibody half-lives (whichever is longer) before Day 1 or expected receipt of any investigation product during the follow-up period, or concurrent participation in another interventional study.

Other Exclusions

Involvement in the planning and/or conduct of the study (applies to both AstraZeneca staff and/or staff at the study site).

Judgment by the investigator that the participant should not participate in the study if the participant was unlikely to comply with study procedures, restrictions, and requirements.

For those of childbearing potential only: currently pregnant (confirmed with a positive pregnancy test) or breastfeeding.

Blood drawn in excess of 450 mL (1 unit) for any reason within 30 days before randomization.

Table S1. Study sites and institutional review board/ethics committee names and locations

**Institution**	**Responsible Institutional Review Board/Ethics Committee**
Huashan Hospital, Fudan University, Shanghai	IRB of Huashan Hospital, Fudan University, Shanghai
Nanfang Hospital, Southern Medical University, Guangzhou	Medical Ethics Committee of Nanfang Hospital of Southern Medical University

Table S2. Demographic and Baseline Characteristics (Safety Analysis Set)

	AZD7442						
Characteristic	300 mg IM (n = 12)	600 mg IM (n = 12)	300 mg IV (n = 12)	600 mg IV (n = 13)	AZD7442 total (n = 49)	Placebo total (n = 11)	All participants (N = 60)
Age (years)	32.9 [8.9]	32.5 [6.0]	31.8 [5.9]	30.9 [5.8]	32.0 [6.6]	31.0 [5.3]	31.8 [6.3]
Age group (years)							
18–44	10 (83.3)	11 (91.7)	12 (100)	13 (100)	46 (93.9)	11 (100)	57 (95.0)
≥45	2 (16.7)	1 (8.3)	0	0	3 (6.1)	0	3 (5.0)
Sex							
Male	9 (75.0)	9 (75.0)	10 (83.3)	9 (69.2)	37 (75.5)	9 (81.8)	46 (76.7)
Female	3 (25.0)	3 (25.0)	2 (16.7)	4 (30.8)	12 (24.5)	2 (18.2)	14 (23.3)
Asian	12 (100)	12 (100)	12 (100)	13 (100)	49 (100)	11 (100)	60 (100)
Weight (kg)[a]	66.4 [5.5]	68.0 [12.7]	61.2 [7.7]	62.1 [7.1]	64.4 [8.9]	67.2 [9.4]	64.9 [9.0]
BMI (kg/m^2),	24.0 [2.3]	23.8 [2.9]	22.4 [2.4]	23.1 [1.8]	23.3 [2.4]	23.9 [2.8]	23.4 [2.5]

BMI, body mass index, IM, intramuscular, IV intravenous; SD, standard deviation. Values are means [SD] or n (%).

[a]For comparison, the mean body weight in the first-in-human global study was 75.5 kg and 72.2 kg for the 300 mg IM and 300 mg IV cohorts, respectively.[4]

Table S3. Overall Summary of AEs by Dose and Administration Route (Safety Analysis Set)

AE category, n[a] (%)	AZD7442 300 mg IM (n = 12)	AZD7442 600 mg IM (n = 12)	AZD7442 300 mg IV (n = 12)	AZD7442 600 mg IV (n = 13)	AZD7442 total (n = 49)	Pooled placebo (n = 11)
AE	9 (75.0)	12 (100)	12 (100)	12 (92.3)	45 (91.8)	9 (81.8)
SAE	0	1 (8.3)	0	1 (7.7)	2 (4.1)	0
Deaths	0	0	0	0	0	0
AE considered related to study drug	2 (16.7)	3 (25.0)	2 (16.7)	2 (15.4)	9 (18.4)	3 (27.3)
Any AESI	0	1 (8.3)	0	0	1 (2.0)	0
AEs by maximum intensity[b]						
Mild	8 (66.7)	11 (91.7)	9 (75.0)	9 (69.2)	37 (75.5)	8 (72.2)
Moderate	1 (8.3)	0	3 (25.0)	3 (23.1)	7 (14.3)	1 (9.1)
Severe	0	1 (8.3)	0	0	1 (2.0)	0
AEs occurring in ≥5% participants in the AZD7442 total group						
COVID-19	5 (41.7)	5 (41.7)	6 (50.0)	8 (61.5)	24 (49.0)	3 (27.3)
Upper respiratory tract infection	3 (25.0)	3 (25.0)	3 (25.0)	3 (23.1)	12 (24.5)	3 (27.3)
Fatigue	2 (16.7)	2 (16.7)	2 (16.7)	1 (7.7)	7 (14.3)	1 (9.1)
Blood creatinine increased	1 (8.3)	0	2 (16.7)	1 (7.7)	4 (8.2)	2 (18.2)
Cough	0	1 (8.3)	1 (8.3)	2 (15.4)	4 (8.2)	1 (9.1)

Headache	0	0	1 (8.3)	3 (23.1)	4 (8.2)	1 (9.1)
Lymphocyte percentage decreased	0	4 (33.3)	0	0	4 (8.2)	0
Neutrophil count increased	0	1 (8.3)	2 (16.7)	1 (7.7)	4 (8.2)	0
Rhinorrhea	0	0	3 (25.0)	1 (7.7)	4 (8.2)	1 (9.1)
Alanine aminotransferase increased	1 (8.3)	1 (8.3)	0	1 (7.7)	3 (6.1)	1 (9.1)
Diarrhea	1 (8.3)	0	1 (8.3)	1 (7.7)	3 (6.1)	0
Neutrophil percentage increased	0	3 (25.0)	0	0	3 (6.1)	0
White blood cells urine positive	1 (8.3)	1 (8.3)	0	1 (7.7)	3 (6.1)	0

AE, adverse event; AESI, AE of special interest; COVID-19, coronavirus disease 2019; IM, intramuscular; IV, intravenous; n, number of participants per treatment group; SAE, serious AE.

[a]n = number of participants with at least 1 event. Participants with multiple events in the same category were counted only once in that category. Participants with events in more than 1 category were counted once in each of those categories. [b]Participants are counted once for the maximum intensity. The table includes AEs (preferred terms in Medical Dictionary for Regulatory Activities version 25.1) with ≥5% incidence in the AZD7442 total group with an onset date/time on or after the date/time of dosing. Table is sorted by decreasing number of participants based on the AZD7442 total group. Included COVID-19 cases were confirmed with a positive antigen or nucleic acid test result.

Table S4. Summary of ADA Responses to AZD7442

ADA category	AZD7442 300 mg IM (N = 12)	AZD7442 600 mg IM (N = 12)	AZD7442 300 mg IV (N = 12)	AZD7442 600 mg IV (N = 13)	AZD7442 Total (N = 49)	Pooled Placebo (N = 11)
ADA prevalence	1 (8.3)	1 (8.3)	1 (8.3)	1 (7.7)	4 (8.2)	2 (18.2)
	320	80	160	640	240	80
	320, 320	80, 80	160, 160	640, 640	80, 640	80, 80
Only baseline positive	0	1 (8.3)	0	0	1 (2.0)	0
	N/A	80	N/A	N/A	80	N/A
	N/A, N/A	80, 80	N/A, N/A	N/A, N/A	80, 80	N/A, N/A
Baseline and post-baseline positive	0	0	0	0	0	2 (18.2)
	N/A	N/A	N/A	N/A	N/A	80
	N/A, N/A	N/A, N/A	N/A, N/A	N/A, N/A	N/A, N/A	80, 80
TE-ADA positive[a]	1 (8.3)	0	1 (8.3)	1 (7.7)	3 (6.1)	0
	320	N/A	160	640	320	N/A
	320, 320	N/A, N/A	160, 160	640, 640	160, 640	N/A, N/A
Treatment-induced ADA positive	1 (8.3)	0	1 (8.3)	1 (7.7)	3 (6.1)	0
	320	N/A	160	640	320	N/A

	320, 320	N/A, N/A	160, 160	640, 640	160, 640	N/A, N/A
Treatment-boosted ADA positive	0	0	0	0	0	0
Non-TE-ADA positive[a]	0	1 (8.3)	0	0	1 (2.0)	2 (18.2)
	N/A	80	N/A	N/A	80	80
	N/A, N/A	80, 80	N/A, N/A	N/A, N/A	80, 80	80, 80
ADA persistently positive[b]	1 (8.3)	0	1 (8.3)	1 (7.7)	3 (6.1)	0
	320	N/A	160	640	320	N/A
	320, 320	N/A, N/A	160, 160	640, 640	160, 640	N/A, N/A

ADA, anti-drug antibody; IM, intramuscular; IV, intravenous; n, represents the number of participants satisfying the conditions of the specified ADA category; N, represents the number of participants per treatment group in each respective column among the ADA-evaluable analysis set; N/A, not applicable; TE, treatment-emergent.

Data are presented as n (%), median, and range of titer for each category (top to bottom). ADA prevalence is defined as ADA positive at baseline and/or post-baseline.

[a]TE-ADA positive to AZD7442 is defined as either tixagevimab and/or cilgavimab is TE-ADA positive. Non–TE-ADA positive is defined as at least one of tixagevimab and cilgavimab is positive, and the positive component(s) is/are non–TE-ADA positive. ADA incidence is the proportion of TE-ADA positive participants in a population.

[b]ADA persistently positive to AZD7442 is defined as either tixagevimab and/or cilgavimab is persistently ADA positive.

Figure S1. CONSORT flow diagram (patient disposition)

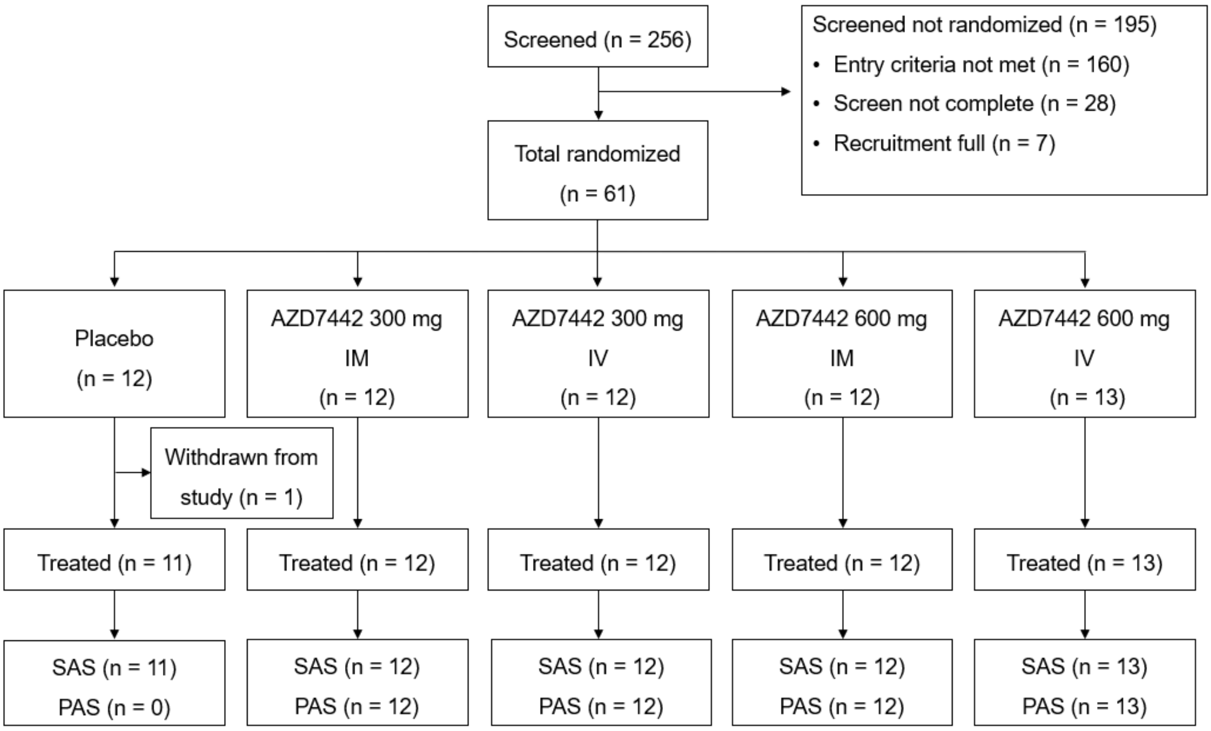

IM, intramuscular; IV, intravenous; PAS, pharmacokinetic analysis set; SAS, safety analysis set.
